# Impact of Era on Acute Cellular Rejection After Lung Transplantation

**DOI:** 10.3389/ti.2025.14534

**Published:** 2025-08-11

**Authors:** Yuriko Terada, Tsuyoshi Takahashi, Michael K. Pasque, Hrishikesh S. Kulkarni, Rodrigo Vazquez-Guillamet, Derek E. Byers, Chad A. Witt, Ruben G. Nava, Benjamin D. Kozower, Bryan F. Meyers, G. Alexander Patterson, Daniel Kreisel, Varun Puri, Ramsey R. Hachem

**Affiliations:** ^1^ Division of Cardiothoracic Surgery, Department of Surgery, Washington University School of Medicine, St. Louis, MO, United States; ^2^ Division of Pulmonary and Critical Care, Department of Medicine, Washington University School of Medicine, St. Louis, MO, United States; ^3^ Division of Pulmonary Medicine, Department of Internal Medicine, University of Utah School of Medicine, Salt Lake City, UT, United States

**Keywords:** lung, transplantation, acute cellular rejection, era, immunosuppression

Dear Editors,

According to the International Society for Heart and Lung Transplantation (ISHLT) registry, survival after lung transplantation has improved significantly over time [1]. In addition, several randomized controlled trials have shown that improvements in maintenance immunosuppression are associated with a lower incidence of acute cellular rejection (ACR) [2, 3]. The ISHLT registry has also shown a modest decrease in the incidence of ACR between 2014 and 2018 (29.0% in 2014, and 27.3% in 2018) [4]. Clearly, ACR remains common despite advances in our understanding of mechanisms of rejection and immunosuppressive therapy. We conducted this study to assess the incidence of ACR and its risk factors over time.

This was a single-center, retrospective cohort study. Between 2009 and 2021, a total of 937 consecutive adult lung transplants were performed at Barnes-Jewish Hospital in St. Louis, Missouri. Among these, 773 patients were included in this study; re-transplants, multi-organ transplants, single lung transplants, and donation after circulatory death-donor transplants were excluded. These patients were stratified into 3 eras based on when they underwent transplantation: Era 1 (2009–2013), Era 2 (2014–2017), and Era 3 (2018–2021). The study protocol was approved by our center’s Institutional Review Board (#202008193). All patients were treated with a triple drug maintenance immunosuppressive regimen after lung transplantation, including a calcineurin inhibitor (tacrolimus or cyclosporine), an antiproliferative agent (mycophenolate mofetil), and corticosteroids. Corticosteroids were initiated on postoperative day 0 at 1 g of methylprednisone daily for 3 days followed by 1 mg/kg prednisone (maximum of 40 mg) with a predetermined taper down to 5 mg by 3 months. Lung transplant recipients undergo surveillance bronchoscopy with transbronchial lung biopsies and bronchoalveolar lavage at 1, 2, 3, 6, and 12 months after transplantation. ACR was scored according to the standard ISHLT criteria [5] and defined as the occurrence of ACR grade ≥ A2 detected at any point during surveillance bronchoscopy within the first year after lung transplant. Cox proportional hazards modeling was used for univariate and multivariate analyses of risk factors for ACR, and all variables with p < 0.05 in univariate analyses were included in multivariate models.

There were increases of anoxia as a donor cause of death from 16.3% in Era 1–27.6% in Era 3 (p = 0.02) and distant donors from 43.3% in Era 1–74.5% in Era 3 (Supplementary Table S1, p < 0.001). In recent eras, patients have been older (median age: 56.0 in Era 1, 59.0 in Era 2, and 61.0 in Era 3, p < 0.001). The use of intraoperative cardiopulmonary bypass (CPB) in recent eras (Era 2 and 3) has decreased in comparison with the early era (Era 1), while the use of intraoperative extracorporeal membrane oxygenation and nitric oxide have increased over time from 0.4% to 36.6% (p < 0.001) and from 65.2% to 92.6% (p < 0.001), respectively. Of note, the proportion of patients who developed primary graft dysfunction grade 3 after lung transplantation has gradually decreased from 34.1% in Era 1–24.0% in Era 2, and 19.8% in Era 3 (p < 0.001). The use of basiliximab for induction immunosuppression increased over time from 73.9% in Era 1–93.0% in Era 3 (p < 0.001). The combination of tacrolimus and mycophenolate mofetil was the most commonly used maintenance immunosuppression regimen at discharge and increased in recent eras (p < 0.001). The incidence of ACR has decreased in recent eras (2014–2017 and 2018–2021) compared to the early era (2009–2013). Freedom from ACR at 1 year was 57.2% in Era 1, 70.1% in Era 2, and 72.2% in Era 3 (Figure 1, p < 0.001).

**FIGURE 1:**
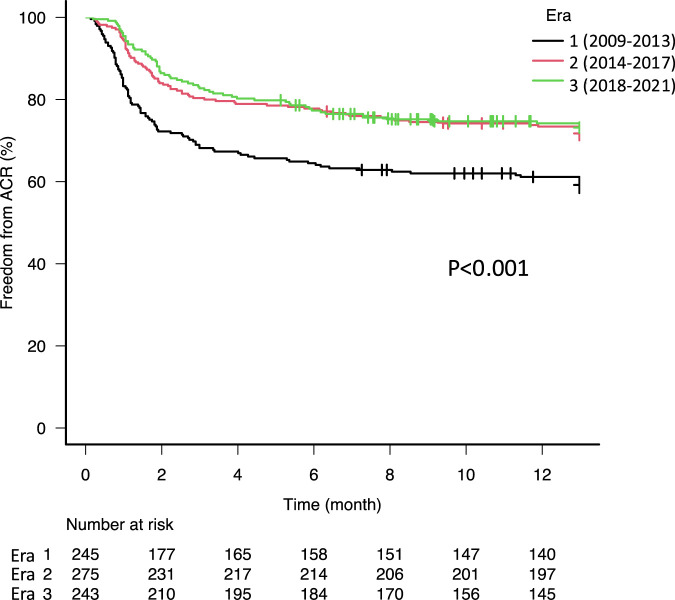
Kaplan-Meier freedom from acute cellular rejection (ACR) curves stratified by era. The 1-year freedom from ACR was significantly higher in era 2 (70.1%) and 3 (72.2%) compared to era 1 (57.2%, p < 0.001).

Multivariate Cox proportional hazards analysis showed that Eras 2 and 3 were associated with a decreased risk of ACR compared to Era 1 (Supplementary Table S2, hazard ratio [HR]: 0.602, 95% confidence interval [CI]: 0.449–0.808, p = 0.002, HR: 0.666, 95% CI: 0.477–0.924, p < 0.001, respectively). Although the number of lung transplants performed in patients with cystic fibrosis has drastically decreased over time (Supplementary Table S1), multivariate analysis revealed that cystic fibrosis was not a significant factor associated with the recent reduction in the incidence of ACR.

Previous studies have shown younger patients have been reported to be at increased risk in multiples studies [6]. Although over 70% of lung recipients are treated with basiliximab and its use has become more commonplace internationally according to the ISHLT Registry [1] the use of basiliximab was not associated with a lower risk of ACR in our study. Indeed, the superiority of basiliximab in comparison with thymoglobulin has not been demonstrated in other studies [7]. In contrast, a number of randomized controlled trials have demonstrated a lower risk of ACR in patients treated with tacrolimus compared to those treated with cyclosporine A [2, 8]. Small case series suggested that mycophenolate mofetil was superior to azathioprine in preventing ACR, but this finding has not been consistent in other studies [9]. Taken together, these data and our results suggest that recipient age and the more frequent use of tacrolimus may contribute to the decreased incidence of ACR in the more recent era.

This study has important limitations inherent to its design. This was a retrospective single-center study. However, the results are consistent with the ISHLT Registry. Nonetheless, it is difficult to make firm conclusions about the impact of the immunosuppressive regimen on ACR because of the retrospective design and categorizing patients based on their maintenance immunosuppression at the time of discharge from the index hospitalization and at 6 months after transplantation. Finally, it is possible that we did not account for other potential factors that may influence the risk of ACR in this analysis, such as human leukocyte antigen mismatches, donor-specific antibodies, antibody-mediated rejection, and respiratory infections following lung transplantation.

Our findings demonstrate an improvement in the incidence of ACR after lung transplantation over time. Recent eras (2014–2017 and 2018–2021) were associated with a significantly lower risk of ACR. There were no significantly independent factors leading to the recent improvement in the incidence of ACR over time in this study. This is likely multifactorial, but we suspect that increasing recipient age, decreasing use of CPB, and the use of tacrolimus and mycophenolate mofetil in recent eras contribute to this lower risk of ACR.

## Data Availability

The raw data supporting the conclusions of this article will be made available by the authors, without undue reservation.

## Abbreviations

ACR, acute cellular rejection; CI, confidence interval; CPB, cardiopulmonary bypass; HR, hazard ratio; ISHLT, International Society for Heart and Lung Transplantation.
