# Supplementary material for: Impact of Era on Acute Cellular Rejection After Lung Transplantation
Source: Transpl Int. 2025 Aug 11;38:14534. doi: 10.3389/ti.2025.14534 (PMC12375509; doi:10.3389/ti.2025.14534)
Supplement: Supplementary file 1 [file Table1.docx]

**Supplementary Table S1: Donor and recipient characteristics**

| **Variable** | **Era 1 (2009-2013)**  **(n=245)** | **Era 2 (2014-2017)**  **(n=275)** | **Era 3 (2018-2021)**  **(n=243)** | **p** |
| --- | --- | --- | --- | --- |
| **Donor information** |  |  |  |  |
| Age, median (IQR), year | 35.0 (24.0-50.0) | 34.0 (23.0-50.0) | 34.0 (24.0-48.0) | 0.75 |
| Sex |  |  |  | 0.09 |
| Male | 159 (64.9%) | 156 (56.7%) | 157 (64.6%) |  |
| Female | 86 (35.1%) | 119 (43.3%) | 86 (35.4%) |  |
| Cause of death |  |  |  | 0.02 |
| Head trauma | 101 (41.2%) | 115 (41.8%) | 102 (42.0%) |  |
| Stroke | 95 (38.8%) | 82 (29.8%) | 63 (25.9%) |  |
| Anoxia | 40 (16.3%) | 70 (25.5%) | 67 (27.6%) |  |
| Other | 9 (3.7%) | 8 (2.9%) | 11 (4.5%) |  |
| Creatinine, median (IQR), mg/dL | 1.0 (0.8-1.2) | 1.0 (0.8-1.3) | 1.1 (0.9-1.6) | <0.001 |
| Best PaO_2_, median (IQR), mmHg | 506 (461-554) | 524 (475-564) | 511 (471-566) | 0.10 |
| Non-local donor | 106 (43.3%) | 114 (41.5%) | 181 (74.5%) | <0.001 |
| **Recipient information** |  |  |  |  |
| **Pre-transplant status** |  |  |  |  |
| Age, median (IQR), year | 56.0 (43.0-62.0) | 59.0 (49.0-66.0) | 61.0 (54.0-65.0) | <0.001 |
| Sex |  |  |  | 0.33 |
| Male | 142 (58.0%) | 158 (57.7%) | 154 (63.4%) |  |
| Female | 103 (42.0%) | 117 (42.3%) | 89 (36.6%) |  |
| Diagnosis |  |  |  | 0.005 |
| ILD | 114 (46.5%) | 148 (53.8%) | 128 (52.7%) |  |
| COPD | 77 (31.4%) | 79 (28.7%) | 84 (34.6%) |  |
| CF | 47 (19.2%) | 43 (15.6%) | 18 (7.4%) |  |
| PVD | 5 (2.0%) | 3 (1.1%) | 11 (4.5%) |  |
| Other | 2 (0.8%) | 2 (0.7%) | 2 (0.8%) |  |
| Smoking history > 20 pack-years | 128 (52.2%) | 144 (52.4%) | 140 (57.6%) | 0.39 |
| CMV mismatch | 104 (42.4%) | 123 (44.7%) | 108 (44.4%) | 0.85 |
| Secondary PAH | 131 / 195 (67.2%) | 157 / 212 (74.1%) | 168 / 215 (78.1%) | 0.04 |
| LAS, median (IQR) | 40.3 (34.3-53.6) | 39.4 (34.7-49.0) | 38.0 (34.1-44.4) | 0.09 |
| Pre-transplant MV | 20 (8.2%) | 21 (7.6%) | 11 (4.5%) | 0.22 |
| Pre-transplant ECMO | 2 (0.8%) | 6 (2.2%) | 10 (4.1%) | 0.054 |
| **Intraoperative status** |  |  |  |  |
| Ischemic time, median (IQR), minute | 269 (225-325) | 243 (198-306) | 309 (267-339) | <0.001 |
| Intraoperative ECMO | 1 (0.4%) | 5 (1.8%) | 89 (36.6%) | <0.001 |
| Intraoperative CPB | 116 (47.3%) | 123 (44.7%) | 32 (13.2%) | <0.001 |
| Nitric oxide | 159 (65.2%) | 212 (77.1%) | 224 (92.6%) | <0.001 |
| Intraoperative pRBC transfusion, median (IQR), unit | 2.0 (2.0-4.0) | 4.0 (2.0-6.0) | 3.0 (2.0-6.0) | <0.001 |
| **Postoperative status** |  |  |  |  |
| Postoperative ECMO | 1 (0.4%) | 8 (2.9%) | 17 (7.0%) | <0.001 |
| Tracheostomy | 39 (15.9%) | 69 (25.1%) | 48 (19.8%) | 0.03 |
| Hospital stay, median (IQR), day | 14 (11-22) | 16 (12-26) | 17 (12-31) | 0.003 |
| PGD grade 3 | 88 (36.1%) | 66 (24.0%) | 48 (19.8%) | <0.001 |
| **Immunosuppression** |  |  |  |  |
| Basiliximab* | 181 (73.9%) | 272 (98.9%) | 226 (93.0%) | <0.001 |
| Thymoglobulin* | 64 (26.1%) | 3 (1.1%) | 17 (7.0%) | <0.001 |
| Tacrolimus+MMF at discharge | 152 (62.0%) | 260 (94.5%) | 217 (89.3%) | <0.001 |
| Tacrolimus+azathioprine at discharge | 88 (35.9%) | 13 (4.7%) | 16 (6.6%) | <0.001 |
| Tacrolimus+MMF at 6 months | 152 (62.0%) | 199 (72.4%) | 164 (67.5%) | 0.04 |
| Tacrolimus+azathioprine at 6 months | 68 (27.8%) | 39 (14.2%) | 31 (12.8%) | <0.001 |
| ACR grade ≥ A2 | 104 (42.4%) | 82 (29.8%) | 66 (27.2%) | 0.001** |

ACR = acute cellular rejection

CF = cystic fibrosis

CMV = cytomegalovirus

COPD = chronic obstructive pulmonary disease

CPB = cardiopulmonary bypass

ECMO = extracorporeal membrane oxygenation

ILD = interstitial lung disease

IQR = interquartile range

LAS = lung allocation score

MMF = mycophenolate mofetil

MV = mechanical ventilation

PAH = pulmonary artery hypertension

PaO_2_ = partial pressure of oxygen

PGD = primary graft dysfunction

pRBC = packed red blood cell

PVD = pulmonary vascular disease

*All recipients during the study period in our institution were given basiliximab or thymoglobulin as an induction therapy.

**A Cox proportional hazards model was used to compare the incidence rates of ACR across different eras.

**Supplementary Table S2: Univariate and multivariate Cox proportional hazards regression analysis for risk factors for ACR ≥ grade 2**

| **Variable** | **Univariate analysis**  **HR (95% CI)** | **p** | **Multivariate analysis**  **HR (95% CI)** | **p** |
| --- | --- | --- | --- | --- |
| Era 1 (2009-2013) | Reference |  | Reference |  |
| Era 2 (2014-2017) | 0.624 (0.466-0.834) | 0.001 | 0.602 (0.449-0.808) | 0.002 |
| Era 3 (2018-2021) | 0.597 (0.438-0.813) | 0.001 | 0.666 (0.477-0.924) | <0.001 |
| **Donor information** |  |  |  |  |
| Age | 0.989 (0.981-1.003) | 0.21 |  |  |
| Male | 1.043 (0.808-1.346) | 0.75 |  |  |
| Cause of death |  |  |  |  |
| Stroke | Reference |  |  |  |
| Head trauma | 1.286 (0.956-1.730) | 0.21 |  |  |
| Anoxia | 1.047 (0.734-1.493) | 0.80 |  |  |
| Other | 1.406 (0.744-2.655) | 0.29 |  |  |
| Best PaO_2_ | 1.000 (0.998-1.001) | 0.71 |  |  |
| **Recipient information** |  |  |  |  |
| **Pre-transplant status** |  |  |  |  |
| Age | 0.990 (0.982-0.998) | 0.02 | 0.993 (0.981-1.005) | 0.26 |
| Male | 0.953 (0.740-1.226) | 0.71 |  |  |
| Diagnosis |  |  |  |  |
| COPD | Reference |  | Reference |  |
| ILD | 1.460 (1.082-1.969) | 0.01 | 0.870 (0.341-2.222) | 0.77 |
| CF | 1.687 (1.143-2.489) | 0.008 | 1.274 (0.774-2.095) | 0.34 |
| PVD | 1.081 (0.435-2.689) | 0.87 | 1.323 (0.967-1.810) | 0.08 |
| Other | 1.301 (0.318-5.317) | 0.71 | 0.971 (0.232-4.061) | 0.97 |
| Smoking history | 0.781 (0.610-1.001) | 0.051 |  |  |
| Secondary PAH | 0.966 (0.710-1.314) | 0.83 |  |  |
| LAS | 1.005 (0.998-1.011) | 0.19 |  |  |
| Pre-transplant MV | 1.098 (0.688-1.752) | 0.70 |  |  |
| Pre-transplant ECMO | 0.642 (0.239-1.724) | 0.38 |  |  |
| **Intraoperative status** |  |  |  |  |
| Ischemic time | 1.000 (0.998-1.002) | 0.77 |  |  |
| Intraoperative ECMO | 0.700 (0.456-1.075) | 0.10 |  |  |
| Intraoperative CPB | 1.457 (1.135-1.870) | 0.003 | 1.271 (0.965-1.675) | 0.09 |
| Nitric oxide | 0.889 (0.665-1.188) | 0.43 |  |  |
| Intraoperative pRBC | 1.051 (0.800-1.382) | 0.72 |  |  |
| **Postoperative status** |  |  |  |  |
| PGD grade 3 | 1.044 (0.790-1.379) | 0.76 |  |  |
| **Immunosuppression** |  |  |  |  |
| Basiliximab | 1.071 (0.713-1.607) | 0.74 |  |  |
| Thymoglobulin | 0.934 (0.622-1.402) | 0.74 |  |  |
| Tacrolimus+MMF at discharge | 1.009 (0.727-1.402) | 0.96 |  |  |
| Tacrolimus+azathioprine at discharge | 1.120 (0.801-1.565) | 0.51 |  |  |
| Tacrolimus+MMF at 6 months | 1.431 (1.079-1.897) | 0.01 | 1.455 (0.430-1.941) | 0.75 |
| Tacrolimus+azathioprine at 6 months | 0.705 (0.493-1.008) | 0.055 |  |  |

ACR = acute cellular rejection

CF = cystic fibrosis

CI = confidence interval

COPD = chronic obstructive pulmonary disease

CPB = cardiopulmonary bypass

ECMO = extracorporeal membrane oxygenation

HR = hazard ratio

ILD = interstitial lung disease

LAS = lung allocation score

MMF = mycophenolate mofetil

MV = mechanical ventilation

pRBC = packed red blood cell

PAH = pulmonary artery hypertension

PaO_2_ = partial pressure of oxygen

PGD = primary graft dysfunction

PVD = pulmonary vascular disease
